# Retroperitoneal lipoma; a benign condition with frightening presentation

**DOI:** 10.1016/j.ijscr.2019.02.044

**Published:** 2019-03-11

**Authors:** Mohammad Hasan M. Al-Ali, Abdulwahid M. Salih, Okba F. Ahmed, Fahmi H. Kakamad, Shvan H. Mohammed, Marwan N. Hassan, Shadi H. Sidiq, Mohammed Q. Mustafa, Kayhan A. Najar, Ismael Y. Abdullah

**Affiliations:** a Al Jamhori Teaching Hospital, Mousl, Iraq; b University of Sulaimani, College of Medicine, Department of Surgery, Sulaimani, Kurdistan Region, Iraq; c Mosul Cardiac Center, Mousl, Iraq; d Kscien Organization, Hamdi Str., Azadi Mall, Sulaimani, Kurdistan Region, Iraq; e Chara Laboratory, Shahedan Street, Kalar, Kurdistan Region, Iraq; f Knowledge University, Erbil, Kurdistan Region, Iraq

**Keywords:** Lipoma, Retroperitoneal, Tumor, Rare

## Abstract

**Introduction:**

lipoma is the most frequent soft tissue tumor in adults. Its occurrence in the retroperitoneal region is extremely rare. The aim of this study is to report a case of retroperitoneal lipoma with a literature review.

**Presentation of case:**

A 34-year-old female presented with abdominal distension and severe back pain for one year duration. She also reported weight loss and constipation. The examination revealed asymmetrical abdominal distension and everted umbilicus. There was a big irregular mass occupying the whole abdomen reaching into the xiphisternum, firm in consistency, smooth surface, and well defined borders. Abdominal ultrasound demonstrated a large retroperitoneal heterogeneous mass. Computed tomography (CT) scan showed a large well defined hyperechoic mass with fibrous septa extending from the left ovary up to the diaphragm. Exploratory laparotomy revealed a giant clearly demarcated fatty tumor adherent to the retroperitoneal fatty tissues. The histopathological examination of the specimen confirmed the diagnosis of the retroperitoneal lipoma.

**Discussion:**

Retroperitoneal lipomas have been reported in various age groups; namely children, middle and old age patients. Based on the characteristic radiological features of the tumor, enormous diagnostic work-up is not justified.

**Conclusion:**

Retroperitoneal lipoma is a very rare variant of lipoma, presents with various signs and symptoms that may be misleading. Radiologic imaging especially CT scan is the diagnostic tool of choice. Surgical resection is the main modality of management.

## Highlights

• Lipoma is the most frequent soft tissue tumor in adults.
• Its occurrence in the retroperitoneal region is extremely rare.
• It presents with various clinical scenarios.
• We present a case of retroperitoneal lipoma with a brief literature review.

## 1 Introduction

Lipoma is a benign proliferation and collection of mature fat cells [1]. It is the most frequent soft tissue tumor in adults [2]. Currently, the exact underlying etiology is not well understood [1]. However, several theories have been proposed like glucose metabolism disturbance, hormone therapy and seeding after resection of a fibroid [2]. Lipomas are classified according to the morphologic characteristics into fibrolipoma, conventional lipoma, angiolipoma, myelolipoma, spindle cell lipoma, and myelolipoma [2]. They are ordinarily occupying the subdermal tissues of the extremities and trunk [1]. Occurrence of lipoma in the retroperitoneal region is an extremely rare finding [3]. In fact, all of the primary retroperitoneal tumors account for only 0.2% of whole body neoplasms. Among these, majority (80%) of the tumors are malignant neoplasm [3]. Retroperitoneal lipoma may arise from the adipose, connective, muscle, lymphatic or nerve tissues, or it may originate from the mesentery, Gerota’s fascia, or urogenital tract [4,5]. They present challenges for diagnosis, management and follow up.

The aim of this study is to report a case of retroperitoneal lipoma in line with SCARE criteria with a literature review [6].

### 1.1 Patient information

A 34-year-old female presented with abdominal distension and severe back pain for one year duration, during which she had been diagnosed and treated as a case of irritable bowel syndrome. She also reported weight loss and constipation. Her past medical history was negative.

### 1.2 Clinical findings

The examination revealed an asymmetrical abdominal distension and everted umbilicus. There was a big irregular mass occupying the whole abdomen reaching into the xiphisternum, firm in consistency, smooth surface and well defined borders. It not attached to the skin and the examiner failed to get above it.

### 1.3 Diagnostic assessment

**Fig. 1:**
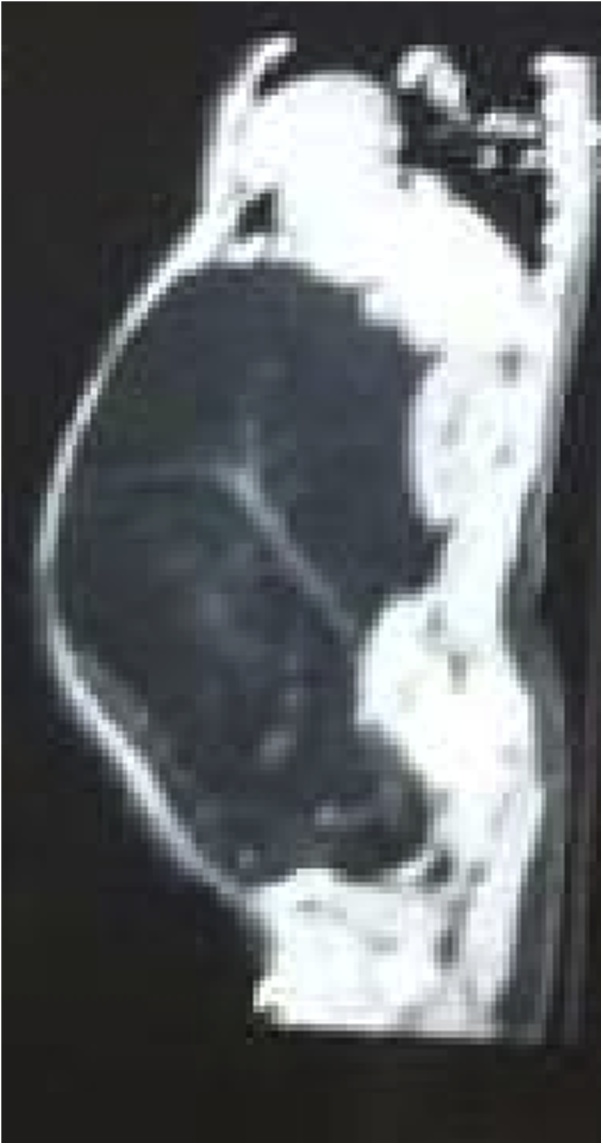
Sagittal computed tomography scan showing fat density mass occupying all abdominal cavity.

**Fig. 2:**
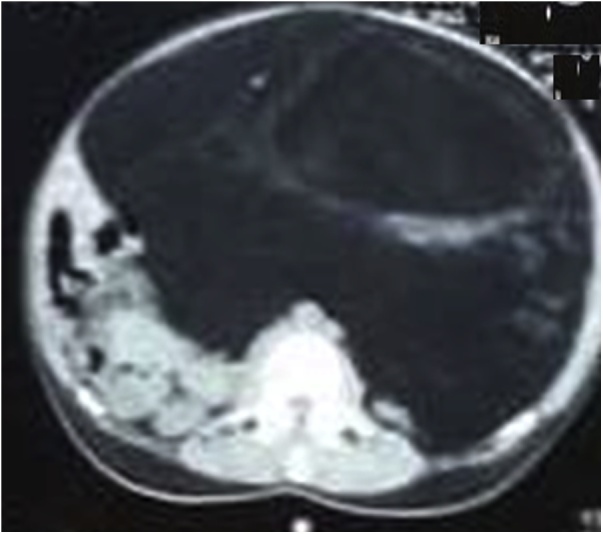
Coronal computed tomography scan showing fat density mass occupying all abdominal cavity.

Complete blood count was normal. Abdominal ultrasound demonstrated a large retroperitoneal heterogeneous mass. The origin of the mass was not clear. It occupied the whole abdominal cavity beyond measurement, displacing the whole abdominal viscera. CT scan showed a large well defined hyperechoic mass with fibrous septa extending from the left ovary up to the diaphragm displacing the bowel to the right and the stomach upward with normal uterus and right ovary, normal size and density of liver and spleen, no evidence of pelvic lymph node enlargement or bony lesions in spine or pelvic bones (Fig. 1, Fig. 2).

### 1.4 Therapeutic intervention

**Fig. 3:**
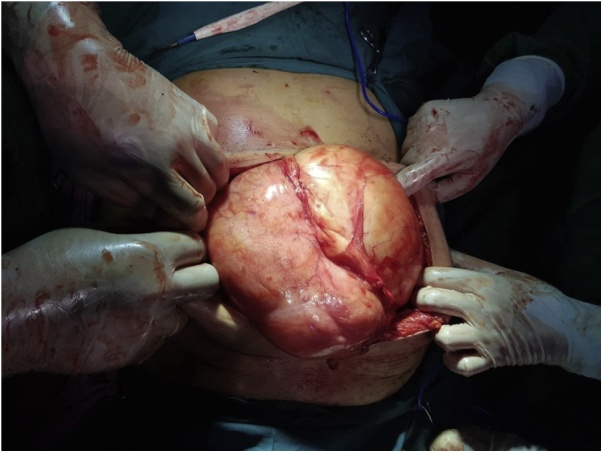
Intraoperative image of the mass.

**Fig. 4:**
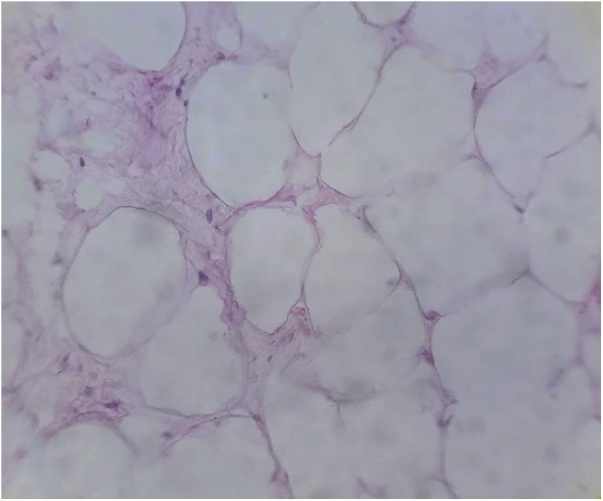
Microscopical picture of the specimen showing multiple mature adipocytes.

After interdisciplinary discussion regarding the management of the case, exploratory laparotomy was done. Intraoperatively, a giant clearly demarcated fatty tumor was found which was adherent to the retroperitoneal fatty tissues extended to the left ovary and measured about 45*48*13 cm (Fig. 3). After resection, it was 12 kilograms. The histopathological examination of the specimen confirmed the diagnosis of retroperitoneal lipoma (Fig. 4).

### 1.5 Follow-up and outcomes

Post operatively, the patient was given a unit of whole blood and kept on intravenous fluid with early mobilization. Bowel motion was observed on the 3^rd^ postoperative day. The patient was discharged on the 6^th^ postoperative day uneventfully. The patient was well six months after the operation and she was free from recurrence.

## 2 Discussion

Retroperitoneal lipomas have been reported in various age groups; namely children, middle and old age patients [4,[7], [8], [9]]. Weniger and associates published their experience with a 73-year-old female presented with recurrent abdominal pain, swelling and obstipation. They opened the patient with suspicion of low grade sarcoma. The histopathological examination of the specimen confirmed the diagnosis of retroperitoneal lipoma [1]. Awais et al reported a 3-year-old boy who presented with progressive abdominal distention and weight gain. Ultrasound guided biopsy revealed normal looking adipocytes without atypia [7]. The current case was a 34-year-old female.

Clinical presentation of retroperitoneal lipoma varies in different reports ranging from abdominal distention to signs and symptoms of sciatica [4,8]. Duran and colleges presented a case complaining of difficulty in walking and leg pain. The patient reported lower back pain radiating to the left lower limb for one year duration. Provisional diagnosis of disc herniation with sciatic nerve compression was assumed although the magnetic resonance imaging failed to support this diagnosis. Later, the patient was diagnosed as a case of retroperitoneal lipoma and relieved by resection [8]. Saito and colleagues reported a case of retroperitoneal lipoma affecting a 65-year-old male presented with intermittent grossly visible hematuria and left flank pain. Excretory urography and ultrasound were normal. Clinically, the diagnosis of nutcracker syndrome was made while CT scan revealed a fat density mass near the left renal pedicle causing dilation of the left renal vein and kinking of left renal artery. The diagnosis of retroperitoneal lipoma was confirmed by the microscopic examination of the specimen. The symptoms subsided after total resection of the mass [9]. The current case was clinically diagnosed and treated as a case of irritable bowel syndrome for about one year.

Based on the characteristic radiological features of the tumor, enormous diagnostic work-up is not justified. However due to rarity of the disease and unawareness of the health care professionals regarding the condition, in most of the time, the provisional diagnosis is misleading [7]. CT scan shows fat density similar to the subcutaneous adipose tissue (Hounsfield units between 65–120) while on T1 weighted MRI, it produces an intense signal [4]. In this case, the size of the lipoma was frightening from the first look as it was very large, displacing almost all of the abdominal organ. Fine needle and tru-cut biopsies were performed by many centers to confirm the diagnosis of the retroperitoneal lipoma before surgical intervention, while others do not recommend preoperative biopsy as the condition is recognizable by imaging, and, in addition to that, the result of the preoperative decision does not affect the type of the management offered to the patient. [1,2,[8], [9], [10]]. The CT scan of the current case was typical for the retroperitoneal lipoma. Histopatholotical examination of the specimen is the gold standard for the confirmation of the diagnosis, although differential low grade liposarcoma is still a problem. Necrosis, atypia, hyperchromatic, pleomorphic and irregular cells and invasion of the surroundings are the differentiating features of liposarcoma [4].

Although the weight of the retroperitoneal lipoma was not documented by most of the authors, the reported weight of the resected specimen is variable ranging from 145 g to 19.5 kg, Table 1. The weight of the tumor in the current case was 12 kg.

**Table 1:** Summary of case reports of retroperitoneal lipomas d in adults since 1970.

Authors, Years [references]	Age	Sex	Tumor size (cm)	Weight (gram)
Weniger et al., 2015 [1]	73	Female	55 × 40 × 10	8950
Duran et al., 2015 [8]	39	Female	6 × 13 × 15	No data
Saito et al., 2013 [9]	65	Male	30 in diameter	No data
Wei et al., 2013 [5]	25	Female	20 × 12 × 10	1650
Chander et al., 2012 [18]	36	Female	13.6 × 11.2 × 9.1	1300
Chander et al., 2012 [18]	65	male	25 × 12	No data
Ukita et al., 2009 [3]	61	Female	15 in diameter	No data
Singh et al., 2009 [12]	65	Male	25 × 12	No data
Singaporewalla et al., 2009 [10]	44	Male	15.6 in diameter	No data
Ida et al., 2008 [13]	65	Male	22 × 14 × 5	No date
Kansakar et al., 2007 [4]	50	Female	30 × 20 × 25	5100
Yildirim et al., 2005 [14]	61	Female	30 × 26 × 17	4390
Drop et al., 2003 [19]	72	Female	12 × 9 × 4	No data
Drop et al., 2003 [19]	60	Female	13 × 12	No data
Martinez et al., 2003 [20]	32	Female	20 × 13 × 10	3400
Raftopoulos et al., 2002 [21]	62	Male	20 × 15 × 10	790
Foa et al., 2002 [22]	52	Male	10.5 × 9.5 × 2	145
Forte et al., 2002 [23]	61	Male	No data	No data
Marshall et al., 2001 [24]	47	Male	No data	4990
Matsubara N. et al., 2000 [25]	65	Male	12 × 13	No data
Acheson et al., 1997 [26]	76	Female	20 × 20 × 12	596
Zhang et al., 1987 [27]	65	Male	50 in diameter	19500
Deppe et al., 1985 [28]	26	Female	11 × 8 x 3	No data
Emmrich et al., 1979 [15]	49	Female	No data	12,500
Mccarthy et al., 1977 [16]	60	male	No data	4990
Mellin et al., 1977 [17]	74	female	No data	9100

Postoperative follow up may not be smooth in all cases of giant retroperitoneal lipoma due to prolonged compression of the abdominal organ (especially the bowel) by the tumor [1,11]. Weniger and associates admitted their case for 18 days post-operatively for management of the paralytic ileus [1].

In conclusion, retroperitoneal lipoma is a very rare variant of lipoma. It presents with various signs and symptoms that may be misleading. Radiologic imaging especially CT scan is the diagnostic tool of choice. Preoperative biopsy (fine needle and tru-cut biopsy) is not mandatory for the diagnosis. Surgical resection is the main modality of management.

## Conflicts of interest

There is no conflict to be declared.

## Sources of funding

No source to be stated.

## Ethical approval

Approval has been taken from Kscien organization for scientific research, no. 51.

## Consent

A written informed consent was obtained from the patient for publication of this case report and accompanying images. A copy of the written consent is available for review by the Editor-in-Chief of this journal on request.

## Author contribution

**Mohammad Hasan M. Al-Ali, Okba F. Ahmed**: preparing the draft with final approval of the manuscript.

**Shvan H. Mohammed, Marwan N. Hassan, Shadi H. Sidiq, Mohammed Q. Mustafa, Kayhan A. Najar, Ismael Y. Abdullah**: revising the draft, reviewing the literature and follow up with final approval of the manuscript.

**Abdulwahid M. Salih**: Revising the manuscript. Final approval of the manuscript.

**Fahmi Hussein Kakamad**: writing the manuscript, reviewing the literature and final approval of the manuscript.

## Registration of research studies

It is a case report, not applicable.

## Guarantor

Fahmi Hussein kakamad.

## Provenance and peer review

Not commissioned, externally peer reviewed.
